# Relapsed Acute Lymphoblastic Leukemia Presenting as Acute Renal Failure

**DOI:** 10.1155/2019/7913027

**Published:** 2019-05-13

**Authors:** Ashley Rose, Samuel Slone, Eric Padron

**Affiliations:** ^1^Internal Medicine, University of South Florida, Tampa, FL 33602, USA; ^2^Malignant Hematology, H. Lee Moffitt Cancer Center and Research Institute, Tampa, FL 33602, USA

## Abstract

Acute lymphoblastic leukemia (ALL) is the second most common acute leukemia in adults. It is an aggressive hematologic neoplasm, with a bimodal age distribution typically presenting in childhood and the 6^th^ decade of life (Terwilliger and Abdul-Hay, 2017). Renal injury in ALL is common and can occur through many different mechanisms, such as prerenal acute kidney injury, acute tubular necrosis, renovascular disease, obstruction, glomerulonephritis, and parenchymal infiltration of tumor cells (Luciano and Brewster, 2014). Infiltration of kidneys by leukemia cells is common; however a resultant injury only occurs in about 1% of patients, and renal failure is even more rare (Luciano and Brewster, 2014). Renal failure due to bilateral infiltration of tumor cells has been reported in only a few cases and is thought to be a poor prognostic indicator (Luciano and Brewster, 2014; Sherief et al., 2015). Biopsy is essential to the diagnosis of renal infiltration of leukemia. We present a case of acute renal failure secondary to bilateral renal infiltration of ALL presenting as the first sign of relapse in a young man.

## 1. Introduction

Acute lymphoblastic leukemia (ALL) is the second most common acute leukemia in adults with 75% of cases developing from hematopoietic precursors of B-cell lineage [1]. Kidney involvement and injury in leukemia can occur in a variety of different ways such as prerenal acute kidney injury (AKI), acute tubular necrosis (ATN), parenchymal infiltration by the tumor cells, renovascular diseases, obstruction, glomerulonephritis, and electrolyte/acid-base disturbances [2]. AKI secondary to infiltration of ALL is rare, with renal failure being even more rare [2]. The following is a case of acute renal failure secondary to diffuse renal involvement by B-cell ALL as the first sign of relapse, successfully treated with immunotherapy and steroids.

## 2. Case Presentation

A 37-year-old Hispanic male with a history of B-cell acute lymphocytic leukemia (ALL) presented to the emergency department with left sided flank pain and hematuria. He was previously treated with multiple lines of therapy including several chemotherapy regimens with relapsed/refractory disease. He underwent CAR-T cell therapy in April 2017 and achieved complete remission. He went on to have a mismatched allogeneic hematopoietic stem cell transplant in August 2017 which was complicated by* E. coli* bacteremia and BK cystitis induced hematuria. Soon after, he presented to clinic with acute renal failure and had a ureteral stent placed for left hydronephrosis. Imaging at that time showed symmetric enlargement and decreased density of the kidneys. Serum BK/adenovirus studies were negative. Urine cytology showed benign urothelial cells. Repeat bone marrow biopsy at that time showed 80% cellularity with 80% lymphoblasts. The patient was started on Inotuzumab in March 2018. Repeat bone marrow biopsy following cycle 1 showed no evidence of residual B-cell ALL.

**Figure 1:**
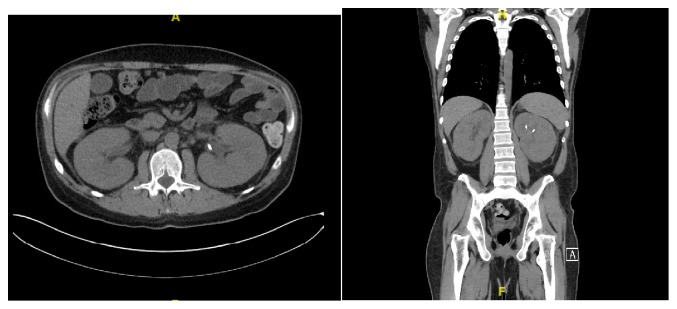
(a), (b) Computed tomography scan showing symmetric enlargement and hypoattenuation of bilateral kidneys with associated fat stranding involving the renal sinuses and perinephric fat. Left sided urinary stent in in place. No hydronephrosis or nephrolithiasis.

**Figure 2:**
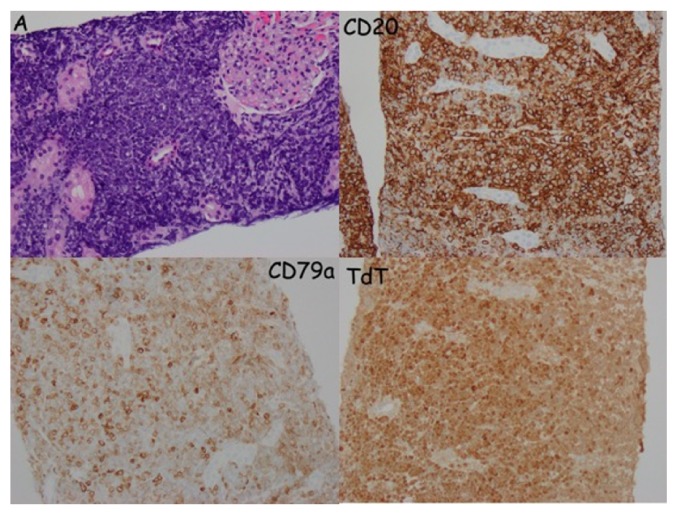
Fine needle aspiration of the kidney.* Top left:* H&E stain shows blue tumor cells infiltrating the kidney, pushing the tubules and glomeruli apart.* Top right:* CD20 marker positivity, which is associated with B-cells.* Bottom left:* CD79 marker positivity, which is associated with B-cells.* Bottom right:* Terminal deoxynucleotidyl transferase (TdT) positivity, often seen in acute lymphoblastic leukemia.

The patient then presented to the emergency department in May 2018 with left sided flank pain and hematuria. Laboratory analysis demonstrated creatinine of 3.9 mg/dL compared to a baseline of 0.6-0.9 mg/dL just 2 weeks earlier. Urinalysis showed negative nitrites, negative leukocyte esterase, >500 protein, 6-10 WBC, 3-5 RBC, and few granular and hyaline casts. Imaging at that time was unchanged from prior imaging, showing symmetric kidney enlargement (Figure 1). Negative work-up included BK viral load, ANCA, anti-GBM antibody, and complement levels. Urine eosinophils were positive. Repeat bone marrow biopsy showed diffuse involvement of B-cell ALL, consistent with relapse. Blood chemistries and uric acid were not consistent with tumor lysis syndrome. Fine needle aspiration of the kidney was performed and demonstrated diffuse invasion of the renal parenchyma by lymphoblasts with positive CD20, CD79, and TdT stains consistent with renal invasion by ALL (Figure 2). The patient had significant oliguria during the first week of hospitalization, with a low daily output of 600mL on day 4. The patient was started on cycle 2 of Inotuzumab as well as Solumedrol 125mg daily for two days, then Prednisone 80mg daily for 5 days followed by a prolonged taper. The patient had significant improvement in hematuria, oliguria, and flank pain following initiation of treatment. His creatinine trended down to 1.5 mg/dL prior to discharge. The patient never required hemodialysis. Unfortunately, several weeks after discharge, the patient presented to the hospital with sepsis secondary to pneumonia and ultimately passed away.

## 3. Discussion

The reported incidence of ALL is estimated to be 1.6/100,000 with a bimodal distribution with the first peak occurring in childhood and the second peak occurring around age 50 [1]. ALL is an aggressive form of cancer with only 30-40% of patient's achieving remission [1]. Malignant lymphoid cells can accumulate in the bone marrow, peripheral blood, or extramedullary sites with the most common locations being lymph nodes, the spleen, and the liver [1].

Acute kidney injury and renal failure are well recognized complications of leukemia. The most common form of kidney injury in leukemia is related to prerenal AKI in the setting of volume depletion (poor PO intake, diarrhea, anorexia, early satiety) [2]. ATN is also common as either an extension of the disease itself (lysozyme-induced tubular necrosis and tumor lysis syndrome) or secondary to a complication commonly seen in the disease process such as sepsis [2]. Glomerular disease can also be seen with hematologic malignancies. In ALL, the most common types of glomerular lesions are minimal change disease (secondary to lysozymuria induced tubular damage) and focal segmental glomerular sclerosis (more common in children) [2]. Kidney infiltration is common in hematologic malignancies and can be seen in 60-90% of patients, with one study of 1200 autopsy cases showing the prevalence of ALL infiltration of the kidney in ~54% of patients at time of death [3].

AKI secondary to infiltration is seen in ~1% of acute leukemia with renal failure secondary to leukemic infiltration being even more rare [2]. In patients with a hematologic malignancy and unexplained renal failure, infiltration should be suspected especially in the setting of widespread disease [4]. Renal failure in these patients is thought to be secondary to acute tubular compression and disruption of the microvasculature leading to ATN [2]. Typical symptoms associated with kidney infiltration secondary to leukemia include hematuria, flank pain, frothy urine [2]. When infiltration is suspected, kidney biopsy is typically pursued as the extent of infiltration can give prognostic information regarding the malignancy as the rate of infiltration parallels the stage and grade of the disease [2]. Leukemic infiltration of the kidneys tends to occur in the late stages of ALL [5]. Renal infiltration has been associated with a poor prognosis [5].

This case represents a rare occurrence of renal failure secondary to leukemic infiltration of bilateral kidneys presenting as a first sign of relapse in this young patient. The patient had already received multiple lines of therapy for relapsed/refractory ALL. Biopsy was obtained proving the etiology of renal failure, and treatment was initiated promptly. The patient had significant improvement in renal function within a few days of therapy, further proving the cause of his renal dysfunction. It is critical to consider leukemic infiltration in a leukemia patient presenting with renal dysfunction. Once common causes are ruled out, biopsy should be obtained and treatment should be initiated. Unfortunately, this patient ultimately passed away due to sepsis.
